# (*E*)-5-[(2-Hy­droxy-5-meth­oxy­benzyl­idene)amino]-1,3,4-thia­diazole-2(3*H*)-thione

**DOI:** 10.1107/S1600536811047362

**Published:** 2011-11-16

**Authors:** Hadi Kargar, Reza Kia, Muhammad Nawaz Tahir

**Affiliations:** a Department of Chemistry, Payame Noor University, PO Box 19395-3697 Tehran, Iran; b X-ray Crystallography Lab., Plasma Physics Research Center, Science and Research Branch, Islamic Azad University, Tehran, Iran; c Department of Chemistry, Science and Research Branch, Islamic Azad University, Tehran, Iran; d Department of Physics, University of Sargodha, Punjab, Pakistan

## Abstract

In the title thione–Schiff base compound, C_10_H_9_N_3_O_2_S_2_, the dihedral angle between the benzene ring and the five-membered ring is 6.69 (8)°. An intra­molecular O—H⋯N hydrogen bond forms an *S*_2_^2^(6) ring. In the crystal, inversion dimers linked by pairs of N—H⋯S inter­actions occur, generating *R*_2_^2^(8) ring motifs. The crystal structure features a S⋯S contact [3.3776 (7) Å], which is significantly shorter than the sum of the van der Waals radii (3.7 Å). The crystal structure also features C—H⋯O and π–π inter­actions [centroid–centroid distances = 3.4636 (9)–3.808 (1) Å].

## Related literature

For standard values of bond lengths, see: Allen *et al.* (1987 ▶). For hydrogen-bond motifs, see: Bernstein *et al.* (1995 ▶). For the biological versatility of thione ligands see, for example: Kumar *et al.* (1988 ▶); Yadav *et al.* (1989 ▶). For a related structure, see: Zhang (2003 ▶). For van der Waals radii, see: Bondi, (1964 ▶).
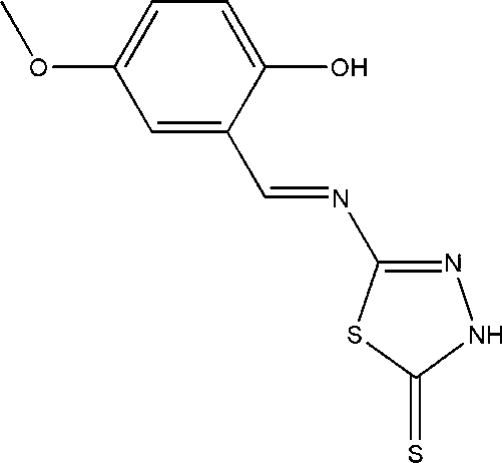


## Experimental

### 

#### Crystal data

C_10_H_9_N_3_O_2_S_2_
*M* *_r_* = 267.32
Triclinic, 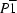

*a* = 6.2266 (2) Å
*b* = 8.0680 (2) Å
*c* = 11.9695 (3) Å
α = 83.027 (2)°
β = 77.993 (1)°
γ = 87.898 (1)°
*V* = 583.76 (3) Å^3^
*Z* = 2
Mo *K*α radiation
μ = 0.45 mm^−1^
*T* = 291 K
0.11 × 0.08 × 0.05 mm

#### Data collection

Bruker SMART APEXII CCD area-detector diffractometer
Absorption correction: multi-scan (*SADABS*; Bruker, 2005 ▶) *T*_min_ = 0.952, *T*_max_ = 0.978
10303 measured reflections
2894 independent reflections
1990 reflections with *I* > 2σ(*I*)
*R*_int_ = 0.030

#### Refinement

*R*[*F* ^2^ > 2σ(*F* ^2^)] = 0.040
*wR*(*F* ^2^) = 0.102
*S* = 1.02
2894 reflections
155 parameters
H-atom parameters constrained
Δρ_max_ = 0.30 e Å^−3^
Δρ_min_ = −0.25 e Å^−3^

### 

Data collection: *APEX2* (Bruker, 2005 ▶); cell refinement: *SAINT* (Bruker, 2005 ▶); data reduction: *SAINT*; program(s) used to solve structure: *SHELXTL* (Sheldrick, 2008 ▶); program(s) used to refine structure: *SHELXTL*; molecular graphics: *SHELXTL*; software used to prepare material for publication: *SHELXTL* and *PLATON* (Spek, 2009 ▶).

## Supplementary Material

Crystal structure: contains datablock(s) global, I. DOI: 10.1107/S1600536811047362/ff2041sup1.cif

Structure factors: contains datablock(s) I. DOI: 10.1107/S1600536811047362/ff2041Isup2.hkl

Supplementary material file. DOI: 10.1107/S1600536811047362/ff2041Isup3.cml

Additional supplementary materials:  crystallographic information; 3D view; checkCIF report

## Figures and Tables

**Table 1:** Hydrogen-bond geometry (Å, °)

*D*—H⋯*A*	*D*—H	H⋯*A*	*D*⋯*A*	*D*—H⋯*A*
O1—H1⋯N1	0.85	1.84	2.616 (2)	151
N3—H3⋯S2^i^	0.80	2.53	3.3163 (15)	169
C2—H2*A*⋯O1^ii^	0.93	2.57	3.481 (2)	167
C3—H3*A*⋯O2^iii^	0.93	2.52	3.442 (3)	172

Symmetry codes: (i) 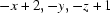; (ii) 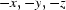; (iii) 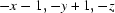.

**Fig. 1.**
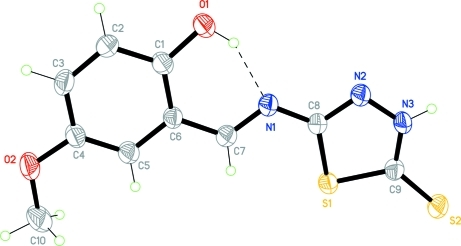
ORTEP plot of the title compound, showing 40% probability displacement ellipsoids. The intramolecular hydrogen bond is drawn as dashed line.

**Fig. 2.**
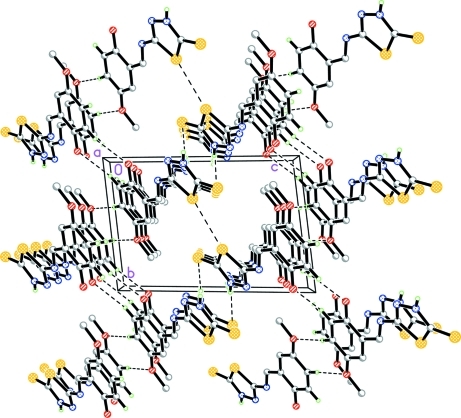
Packing diagram of the title compound viewed down the a-axis. Hydrogen bonds and S···S contacts are drawn as dashed lines.

## supplementary crystallographic information

### Comment

Compounds incorporating a thiadiazole ring have attracted much attention due to their biological activity (Kumar *et al.*, 1988; Yadav *et al.*, 1989). Here we report the crystal structure of a new Schiff base compound containing a thiadiazol ring system.

The asymmetric unit of the title compound, Fig. 1, comprises a thione-Schiff base ligand. The bond lengths (Allen *et al.*, 1987) and angles are within the normal ranges and are comparable to the related structure (Zhang, 2003).

The dihedral angle between the benzene ring and the five-membered ring is 6.69 (8)°. An intramolecular O—H···N hydrogen bond makes S_2_^2^(6) ring motif. Intermolecular N—H···S interactions link neighboring molecules into individual dimers with R_2_^2^(8) ring motifs (Bernstein *et al.*,1995). The interesting feature of the crystal structure is a short S(1)···S(1)^i^ [3.3776 (7)Å; (i) 1 - x,1 - y,1 - z ] contact which is significantly shorter than the sum of the Van der Waals radius of S atoms (Bondi, 1964). The crystal structure is stabilized by the intermolecular C—H···O, and π-π interactions [*Cg1*···*Cg1*^iv^ = 3.4636 (9)Å, (iv) -*1 - x*, *-y*, 1 -*z*; *Cg*1···*Cg*2^v^ = 3.5242 (10)Å, (v) *1 + x*, *y*, *z*; *Cg2*···*Cg2*^vi^ = 3.808 (1)Å, (vi) *-x*, 1 - *y*, *-z Cg1* and *Cg2* are centroids of S(1)/C(8)/N(2)/N(3)/C(9) and C1–C6 rings, respectively.

### Experimental

The title compound was synthesized by adding 5-methoxy-salicylaldehyde (1 mmol) to a solution of 5-aminothiophene-2-thiol (1 mmol) in ethanol (30 ml). The mixture was refluxed with stirring for half an hour. The resultant solution was filtered. Yellow single crystals of the title compound suitable for *X*-ray structure determination were recrystallized from ethanol by slow evaporation of the solvents at room temperature over several days.

### Refinement

All hydrogen atoms were positioned geometrically with C—H = 0.93-0.96 Å and included in a riding model approximation with U_iso_ (H) = 1.2 or 1.5 U_eq_ (C). A rotating group model was applied to the methyl group.

### Crystal data

**Table:**

C_10_H_9_N_3_O_2_S_2_	*Z* = 2
*M**_r_* = 267.32	*F*(000) = 276
Triclinic, *P*1	*D*_x_ = 1.521 Mg m^−^^3^
Hall symbol: -P 1	Mo *K*α radiation, λ = 0.71073 Å
*a* = 6.2266 (2) Å	Cell parameters from 2525 reflections
*b* = 8.0680 (2) Å	θ = 2.5–27.4°
*c* = 11.9695 (3) Å	µ = 0.45 mm^−^^1^
α = 83.027 (2)°	*T* = 291 K
β = 77.993 (1)°	Block, yellow
γ = 87.898 (1)°	0.11 × 0.08 × 0.05 mm
*V* = 583.76 (3) Å^3^	

### Data collection

**Table:**

Bruker SMART APEXII CCD area-detector diffractometer	2894 independent reflections
Radiation source: fine-focus sealed tube	1990 reflections with *I* > 2σ(*I*)
graphite	*R*_int_ = 0.030
φ and ω scans	θ_max_ = 28.4°, θ_min_ = 2.9°
Absorption correction: multi-scan (*SADABS*; Bruker, 2005)	*h* = −8→5
*T*_min_ = 0.952, *T*_max_ = 0.978	*k* = −10→10
10303 measured reflections	*l* = −15→15

### Refinement

**Table:**

Refinement on *F*^2^	Primary atom site location: structure-invariant direct methods
Least-squares matrix: full	Secondary atom site location: difference Fourier map
*R*[*F*^2^ > 2σ(*F*^2^)] = 0.040	Hydrogen site location: inferred from neighbouring sites
*wR*(*F*^2^) = 0.102	H-atom parameters constrained
*S* = 1.02	*w* = 1/[σ^2^(*F*_o_^2^) + (0.0525*P*)^2^] where *P* = (*F*_o_^2^ + 2*F*_c_^2^)/3
2894 reflections	(Δ/σ)_max_ = 0.001
155 parameters	Δρ_max_ = 0.30 e Å^−^^3^
0 restraints	Δρ_min_ = −0.25 e Å^−^^3^

### Special details

**Table:**

Geometry. All esds (except the esd in the dihedral angle between two l.s. planes) are estimated using the full covariance matrix. The cell esds are taken into account individually in the estimation of esds in distances, angles and torsion angles; correlations between esds in cell parameters are only used when they are defined by crystal symmetry. An approximate (isotropic) treatment of cell esds is used for estimating esds involving l.s. planes.
Refinement. Refinement of F^2^ against ALL reflections. The weighted R-factor wR and goodness of fit S are based on F^2^, conventional R-factors R are based on F, with F set to zero for negative F^2^. The threshold expression of F^2^ > 2sigma(F^2^) is used only for calculating R-factors(gt) etc. and is not relevant to the choice of reflections for refinement. R-factors based on F^2^ are statistically about twice as large as those based on F, and R- factors based on ALL data will be even larger.

### Fractional atomic coordinates and isotropic or equivalent isotropic displacement parameters (Å^2^)

**Table:**

	*x*	*y*	*z*	*U*_iso_*/*U*_eq_	
S1	0.51725 (8)	0.31804 (6)	0.44207 (4)	0.04448 (16)	
S2	0.89337 (8)	0.25182 (7)	0.56684 (5)	0.05370 (18)	
O1	0.1438 (3)	0.05675 (17)	0.13460 (13)	0.0639 (4)	
H1	0.2279	0.0646	0.1813	0.096*	
O2	−0.3796 (3)	0.62067 (18)	0.12072 (13)	0.0651 (4)	
N1	0.3359 (2)	0.18322 (18)	0.27908 (12)	0.0401 (4)	
N2	0.6290 (2)	0.05119 (18)	0.34526 (12)	0.0409 (4)	
N3	0.7659 (2)	0.07198 (18)	0.41793 (12)	0.0402 (4)	
H3	0.8576	0.0018	0.4258	0.048*	
C1	0.0238 (3)	0.2001 (2)	0.13354 (15)	0.0421 (4)	
C2	−0.1342 (3)	0.2186 (2)	0.06674 (16)	0.0457 (5)	
H2A	−0.1529	0.1347	0.0229	0.055*	
C3	−0.2630 (3)	0.3596 (2)	0.06470 (15)	0.0449 (5)	
H3A	−0.3691	0.3701	0.0198	0.054*	
C4	−0.2373 (3)	0.4871 (2)	0.12883 (15)	0.0418 (4)	
C5	−0.0803 (3)	0.4727 (2)	0.19435 (14)	0.0404 (4)	
H5	−0.0620	0.5587	0.2366	0.049*	
C6	0.0536 (3)	0.3286 (2)	0.19832 (14)	0.0352 (4)	
C7	0.2144 (3)	0.3154 (2)	0.26892 (14)	0.0385 (4)	
H7A	0.2320	0.4045	0.3086	0.046*	
C8	0.4892 (3)	0.1745 (2)	0.34711 (14)	0.0364 (4)	
C9	0.7411 (3)	0.2037 (2)	0.47685 (15)	0.0391 (4)	
C10	−0.3578 (4)	0.7562 (3)	0.1826 (2)	0.0667 (6)	
H10A	−0.3879	0.7189	0.2635	0.100*	
H10B	−0.4599	0.8434	0.1666	0.100*	
H10C	−0.2109	0.7979	0.1596	0.100*	

### Atomic displacement parameters (Å^2^)

**Table:**

	*U*^11^	*U*^22^	*U*^33^	*U*^12^	*U*^13^	*U*^23^
S1	0.0469 (3)	0.0408 (3)	0.0532 (3)	0.0106 (2)	−0.0243 (2)	−0.0142 (2)
S2	0.0522 (3)	0.0568 (3)	0.0638 (3)	0.0094 (2)	−0.0324 (3)	−0.0208 (3)
O1	0.0781 (10)	0.0504 (9)	0.0822 (10)	0.0294 (8)	−0.0512 (8)	−0.0319 (8)
O2	0.0684 (10)	0.0569 (9)	0.0849 (11)	0.0316 (7)	−0.0458 (8)	−0.0255 (8)
N1	0.0404 (8)	0.0411 (9)	0.0433 (8)	0.0049 (7)	−0.0180 (7)	−0.0081 (7)
N2	0.0412 (8)	0.0420 (9)	0.0451 (8)	0.0065 (7)	−0.0191 (7)	−0.0108 (7)
N3	0.0392 (8)	0.0391 (8)	0.0469 (8)	0.0090 (6)	−0.0198 (7)	−0.0073 (7)
C1	0.0491 (11)	0.0382 (10)	0.0424 (10)	0.0097 (8)	−0.0166 (8)	−0.0085 (8)
C2	0.0554 (12)	0.0430 (11)	0.0461 (10)	0.0054 (9)	−0.0239 (9)	−0.0134 (9)
C3	0.0453 (10)	0.0530 (12)	0.0418 (10)	0.0037 (9)	−0.0218 (8)	−0.0057 (9)
C4	0.0440 (10)	0.0409 (10)	0.0422 (10)	0.0109 (8)	−0.0139 (8)	−0.0062 (8)
C5	0.0442 (10)	0.0392 (10)	0.0406 (9)	0.0063 (8)	−0.0137 (8)	−0.0091 (8)
C6	0.0361 (9)	0.0372 (9)	0.0342 (8)	0.0036 (7)	−0.0119 (7)	−0.0049 (7)
C7	0.0395 (10)	0.0398 (10)	0.0385 (9)	0.0012 (8)	−0.0125 (8)	−0.0068 (8)
C8	0.0361 (9)	0.0357 (9)	0.0397 (9)	0.0022 (8)	−0.0135 (8)	−0.0039 (8)
C9	0.0367 (9)	0.0401 (10)	0.0415 (9)	0.0011 (8)	−0.0119 (8)	−0.0028 (8)
C10	0.0740 (15)	0.0496 (13)	0.0833 (16)	0.0266 (11)	−0.0277 (13)	−0.0228 (12)

### Geometric parameters (Å, °)

**Table:**

S1—C9	1.7383 (18)	C1—C6	1.407 (2)
S1—C8	1.7550 (17)	C2—C3	1.368 (3)
S2—C9	1.6624 (19)	C2—H2A	0.9300
O1—C1	1.354 (2)	C3—C4	1.389 (3)
O1—H1	0.8513	C3—H3A	0.9300
O2—C4	1.377 (2)	C4—C5	1.368 (2)
O2—C10	1.418 (3)	C5—C6	1.408 (2)
N1—C7	1.293 (2)	C5—H5	0.9300
N1—C8	1.373 (2)	C6—C7	1.432 (2)
N2—C8	1.297 (2)	C7—H7A	0.9300
N2—N3	1.366 (2)	C10—H10A	0.9600
N3—C9	1.332 (2)	C10—H10B	0.9600
N3—H3	0.8004	C10—H10C	0.9600
C1—C2	1.385 (3)		
			
C9—S1—C8	89.57 (8)	C4—C5—H5	119.8
C1—O1—H1	105.8	C6—C5—H5	119.8
C4—O2—C10	117.48 (15)	C1—C6—C5	118.89 (16)
C7—N1—C8	120.57 (15)	C1—C6—C7	121.70 (16)
C8—N2—N3	109.05 (15)	C5—C6—C7	119.40 (16)
C9—N3—N2	120.21 (15)	N1—C7—C6	121.91 (17)
C9—N3—H3	121.3	N1—C7—H7A	119.0
N2—N3—H3	118.5	C6—C7—H7A	119.0
O1—C1—C2	118.24 (16)	N2—C8—N1	120.02 (16)
O1—C1—C6	122.23 (17)	N2—C8—S1	114.13 (13)
C2—C1—C6	119.53 (17)	N1—C8—S1	125.85 (13)
C3—C2—C1	120.48 (17)	N3—C9—S2	127.15 (14)
C3—C2—H2A	119.8	N3—C9—S1	107.00 (13)
C1—C2—H2A	119.8	S2—C9—S1	125.86 (11)
C2—C3—C4	120.82 (17)	O2—C10—H10A	109.5
C2—C3—H3A	119.6	O2—C10—H10B	109.5
C4—C3—H3A	119.6	H10A—C10—H10B	109.5
C5—C4—O2	125.16 (16)	O2—C10—H10C	109.5
C5—C4—C3	119.82 (17)	H10A—C10—H10C	109.5
O2—C4—C3	115.02 (16)	H10B—C10—H10C	109.5
C4—C5—C6	120.46 (17)		
			
C8—N2—N3—C9	0.3 (2)	C4—C5—C6—C7	−179.03 (16)
O1—C1—C2—C3	178.58 (18)	C8—N1—C7—C6	179.75 (15)
C6—C1—C2—C3	−0.9 (3)	C1—C6—C7—N1	−1.9 (3)
C1—C2—C3—C4	0.4 (3)	C5—C6—C7—N1	177.34 (16)
C10—O2—C4—C5	1.9 (3)	N3—N2—C8—N1	178.84 (14)
C10—O2—C4—C3	−178.74 (19)	N3—N2—C8—S1	−1.70 (19)
C2—C3—C4—C5	0.5 (3)	C7—N1—C8—N2	−171.14 (17)
C2—C3—C4—O2	−178.95 (18)	C7—N1—C8—S1	9.5 (2)
O2—C4—C5—C6	178.60 (17)	C9—S1—C8—N2	2.05 (14)
C3—C4—C5—C6	−0.8 (3)	C9—S1—C8—N1	−178.53 (16)
O1—C1—C6—C5	−178.85 (17)	N2—N3—C9—S2	−178.78 (13)
C2—C1—C6—C5	0.6 (3)	N2—N3—C9—S1	1.2 (2)
O1—C1—C6—C7	0.4 (3)	C8—S1—C9—N3	−1.69 (13)
C2—C1—C6—C7	179.87 (17)	C8—S1—C9—S2	178.29 (13)
C4—C5—C6—C1	0.2 (3)		

### Hydrogen-bond geometry (Å, °)

**Table:**

*D*—H···*A*	*D*—H	H···*A*	*D*···*A*	*D*—H···*A*
O1—H1···N1	0.85	1.84	2.616 (2)	151
N3—H3···S2^i^	0.80	2.53	3.3163 (15)	169
C2—H2A···O1^ii^	0.93	2.57	3.481 (2)	167
C3—H3A···O2^iii^	0.93	2.52	3.442 (3)	172

Symmetry codes: (i) −*x*+2, −*y*, −*z*+1; (ii) −*x*, −*y*, −*z*; (iii) −*x*−1, −*y*+1, −*z*.
